# Childhood growth of singletons conceived following intracytoplasmic sperm injection – irrelevance of gonadotropin stimulation

**DOI:** 10.3389/frph.2024.1453697

**Published:** 2024-09-23

**Authors:** M. A. Minger, G. Sommer, V. R. Mitter, L. A. Purtschert, M. von Wolff, A. S. Kohl Schwartz

**Affiliations:** ^1^ Division of Gynecological Endocrinology and Reproductive Medicine, Department of Gynecology, Bern University Hospital, University of Bern, Inselspital, Bern, Switzerland; ^2^ Department of General Surgery, Cantonal Hospital of Graubünden, Chur, Switzerland; ^3^ Division of Paediatric Endocrinology, Diabetology and Metabolism, Department of Paediatrics, Bern University Hospital, University of Bern, Inselspital, Bern, Switzerland; ^4^ Department of BioMedical Research, University of Bern, Bern, Switzerland; ^5^ Institute of Social and Preventive Medicine, University of Bern, Bern, Switzerland; ^6^ Applied Research and Development, Division of Midwifery, Department of Health Professions, Bern University of Applied Sciences, Bern, Switzerland; ^7^ Department of Pulmonology, Cantonal Hospital of Lucerne, Lucerne, Switzerland; ^8^ Division of Reproductive Medicine and Gynecological Endocrinology, Women’s Hospital, University Hospital of Lucerne, Lucerne, Switzerland

**Keywords:** intracytoplasmic sperm injection, natural cycle, gonadotropin stimulation, assisted reproductive technology, birth weight, growth, head circumference

## Abstract

**Background:**

In conventional, gonadotropin stimulated, in vitro fertilization or intracytoplasmic sperm injection (c-IVF/ICSI) growth and development of multiple follicles is induced by gonadotropins, combined with gonadotropin-releasing hormone agonist or antagonist. In recent studies, singletons conceived after c-IVF/ICSI cycles had lower birth weight not only than spontaneously conceived children but also children born after unstimulated natural IVF/ICSI cycles (NC-IVF/ICSI). Lower birth weight is associated with a catch-up growth within the first years of life. Following the Barker hypothesis accelerated growth has been associated with a higher risk of cardiovascular diseases later in life. The aim of the study is to assess, if children conceived with NC-IVF/ICSI have a higher birthweight and therefore do not show a catch-up growth within the first two years. Therefore, we assume that children born after NC-IVF/ICSI have a better long-term cardiometabolic risk profile. Whether the weight- and height gain is comparable to spontaneously conceived children is unknown, since to our knowledge we are the first study to investigate the longitudinal growth of children born after unstimulated natural cycle ICSI (NC-ICSI).

**Material and methods:**

We conducted a single-center, prospective cohort study (2010-2017) including children (*n* = 139) born after NC-ICSI or c-ICSI treatment. Growth parameters up to 24 months were collected. Standard deviation scores based on growth references were calculated.

**Results:**

The study included 98 children in the NC-ICSI and 41 children in the c-ICSI group. The median birth weight in NC-ICSI children was 3.4 kg [0.1 standard deviation score (SDS)] compared to 3.3 kg (−0.3 SDS) in c-ICSI children (*p* = 0.61). Median length at birth was 50 cm in both groups (NC-ICSI (−0.5 SDS), c-ICSI children (−0.8 SDS), *p* = 0.48). At age 24 months, median weight in NC-ICSI children was 12.2 kg (0.3 SDS) versus 12.2 kg (0.2 SDS) in c-ICSI children (*p* = 0.82) and median length 87.5 cm (0.1 SDS) versus 88.0 cm (0.4 SDS) (*p* = 0.43).

**Conclusion:**

We found no difference in growth between children conceived after stimulated and unstimulated ICSI. Growth parameters of both treatment groups did not differ from Swiss national growth references (*N* = 8500). One of the main limitations of our study was the small sample size (*N* = 139) of complete data sets over time and the high drop-out rate of 49% (68/139). Nevertheless, with the increasing number of children born after IVF/ICSI every year it is of immense importance to search for possibilities to reduce their long-term cardiometabolic risk and we want our data to contribute to this discussion.

## 1 Introduction

Growth is a critical marker for monitoring health and development from early childhood through adolescence, utilized routinely in pediatric practice (1). Abnormal growth patterns, such as a flattening growth curve, can indicate undernutrition or early manifestation of chronic diseases (2). Measurements should include head circumference, a simple and cost-effective metric for assessing central nervous system development (3). In our article growth is defined as increasing weight, height and head circumference.

Children born following assisted reproductive technology (ART) tend to have a lower birth weight (LBW), lower gestational age and are at higher risk for being born small for gestational age (SGA) compared to spontaneously conceived children (4). Following the Barker hypothesis LBW is linked with a catch-up growth within the first years of life. This catch-up growth during early postnatal life increased long-term cardiovascular risks in adulthood (5–7). However, if catch-up growth in ART children is equally associated with a worse long term cardiovascular risk remains unclear. ART involves many factors that might be associated with a worse perinatal outcome such as: subfertility of parents (8–10), ovarian stimulation with gonadotropins (11), number of oocytes retrieved (12) and laboratory procedures like embryo culture (13). Results of studies comparing fertility treatments such as natural cycle *in vitro* fertilization or intracytoplasmic sperm injection (NC-IVF/ICSI) to conventional gonadotropin stimulated treatments (c-IVF/ICSI) show higher birth weight (11, 14, 15) and lower risk for being born SGA in NC-IVF/ICSI children (14, 15).

This leads to the assumption that children born after NC-IVF/ICSI show weight- and height gain more comparable with spontaneously conceived children leading to a more preferable long term cardiovascular risk profile. With our study, we try to address the question, whether gonadotropin stimulation is the accountable cause for the worse perinatal outcome in c-IVF/ICSI children leading to a lower birth weight and following catch-up growth within the first two year of life.

The aim of our study is to analyze the impact of gonadotropin stimulation on early childhood height-, weight and head circumference gain of children born after c-IVF/ICSI and NC-IVF/ICSI. Within the last years, many different ART techniques have evolved, and the number of children born after ART have largely increased. Therefore, it is crucial to critically analyse and compare different ART techniques with the goal to optimize not only pregnancy rates but also long-term health for ART offspring.

## 2 Methods

### 2.1 Study population

We conducted a single-center cohort study at the University Hospital of Bern, Switzerland to compare weight and height gain as well as head circumference diameter of children born after stimulated and non-stimulated ICSI treatments as a primary focus. The observational period was from birth up to two years of age. The secondary focus was the comparison to a standard control population of children. This is a follow-up study of a subgroup from the Bern IVF Cohort. The data collection of this cohort Bern IVF Cohort was as described before by our group (15, 16). When the children reached the age of two years the parents of all singleton live births born following ICSI treatment between 11/2010 and 12/2017 (before blastocyst culture became legal in Switzerland) were asked to provide information on auxologic data, breastfeeding duration and health (chronic diseases) of their children until age two (*N* = 254). Written informed consent to participate in this study was obtained from the parents. Exclusion criteria were thawed frozen embryos, oocyte- or sperm donation, multiple pregnancies, refusal to participate in research or mild ovarian stimulation schemes (with daily gonadotropin doses below 150 IE/d). To be included in our study, at least five growth measurements within the first two years of life were required (*N* = 139/254 (55%) (NC-ICSI: 98/139 (71%), c-ICSI: 41/139 (29%)).

### 2.2 Ethical approval

Our study was approved by the ﻿cantonal ethical committee of Bern (KEK Bern, 397/15), Switzerland, on the 26th of January 2016 and was amended in August 2018.

### 2.3 Reproductive treatment

Women with a regular menstrual cycle (26–32 days) could choose their preferred treatment, either NC-ICSI or c-ICSI.

NC-ICSI treatment was performed according to short agonist or standard antagonist protocols as previously described (17). The NC-ICSI treatment group included women with monofollicular cycles without any gonadotropin stimulation. Follicles were monitored by ultrasound and analysis of Estradiol (E2) and luteinizing hormone (LH) concentrations. At times, a low-dose of clomiphene citrate (25 mg per day) or ibuprofen was added to reduce the risk of preterm ovulation (18, 19). As soon as follicular maturity was achieved (follicular diameter ≥ 16 mm and E2 concentrations were estimated ≥ 800 pmol/l), a trigger shot of 5,000 IU human chorionic gonadotropin (hCG) for ovulation induction was applied. After 36 h, follicle aspiration was performed using a 19G single lumen needle.

In both types of treatment, fertilization was achieved by ICSI. Both groups had identical culture conditions; in 2010, the embryos were cultured in global® (Cooper Surgical) and in the following years, in global® total® LP (Cooper Surgical). Our study group included all women with pregnancies resulting from single or double cleavage stage embryo transfer ending in a singleton live birth.

### 2.4 Control population

As a control population we used the Swiss national growth references, recommended by the Swiss Society of Pediatrics (20).

### 2.5 Data collection

Data on parents, treatment cycles, pregnancies and perinatal outcomes was collected from medical records as previously described (15, 16, 21). When the child reached age two, the parents received a follow-up questionnaire to complement their child's health and growth parameters. This included height, weight and head circumference at 0, 1, 2, 3, 6, 12,18 and 24 months. We also asked for chronic diseases (including gastrointestinal (coeliac disease), immunologic (strong allergies) dermatologic (neurodermatitis, psoriasis) as well as for height and weight of the parents. They were also asked to send a copy of the child health booklet, where pediatricians note anthropometric measurements from birth to adolescence at each routine health check-up (22). We contacted the parents by phone several times in case we did not receive the questionnaire or the copies of the booklet within a month. Perinatal outcomes were defined as follows: Low birth weight (<2,500 grams) preterm birth (<37 gestational weeks) and very preterm birth (<32 gestational weeks). SGA was defined as a weight per gestational age under the fifth percentile (23). All parameters were electronically recorded using Redcap electronic data (Redcap 8.5.19 Vanderbilt University, Nashville, USA) capture tools hosted at the Clinical Trials Unit, University of Bern (24).

### 2.6 Covariates

We assessed several parental characteristics that could influence our outcome: maternal age (continuous), ethnicity (European, non-European, unknown), educational level (university diploma (tertiary level) > at least an apprenticeship diploma (secondary level) > lower educational level (primary level)), pre-pregnancy body mass index (BMI) (continuous), and chronic disease. Duration of subfertility (<1 year, 1–2 years, >2 years), parity (first, second or further) smoking during pregnancy (yes, no, unknown), reason for infertility and complications during pregnancy (both categorical). Maternal chronic disease included arterial hypertension, thyroid disease, neurodermitis or asthma. Parental chronic disease included arterial hypertension, thyroid disease, neurodermitis or asthma. We also examined age, height, weight and ethnicity of the father.

### 2.7 Statistical analysis

Standard deviations scores (SDS) of the anthropometric parameters weight, length, head circumference, and BMI at the age of one, two, four, six, twelve and 24 months were calculated with the LMS method (Lambda for the skew, Mu for the median, and Sigma for the generalized coefficient of variation) (25) based on the Swiss national growth references (20). We calculated BMI as a measure of the proportion of children's length and height with the formula BMI = weight in kg/height in m^2^.

To account for rapid gain in auxological characteristics during the first months of infancy, we included only examinations measured before or after ten days from the exact age of one, two, four, or six months, respectively. After that, we rounded all measurement time points down to the respective month, e.g., measurements at age 12 months and 25 days were assigned to have been at 12 months. A clinician (MM) interpolated missing data at one of the designated time points on the basis of the Swiss national growth references (20) if all four of the following criteria applied: (1) measurements of at least six time points were available; (2) no more than two consecutive visits were missing; 3) a measurement before and after the missing time point or points was available and (4) growth trajectories did not cross more than one of the following percentiles: the third, tenth, 25th, 50th, 75th, 90th or 97th percentile. We calculated gain in anthropometric parameters in early infancy (﻿SDS at two months of age minus SDS at birth), in late infancy (SDS at twelve months minus SDS at two months) and in early childhood (SDS at 24 months minus SDS at twelve months) similar to Ceelen et al. (26). Further, we did a sensitivity analysis, in which we excluded children born preterm and very preterm.

We compared characteristics of parents and children and anthropometric parameters between NC-ICSI and c-ICSI using Kruskal-Wallis tests for categorical variables and Wilcoxon rank-sum tests for continuous variables. The statistics were analyzed using the statistical software Stata (Version 16, Stata Corporation, Austin, Texas). *P* values <0.05 were considered significant.

## 3 Results

### 3.1 Study population

Between November 2010 and December 2017 there were 254 singleton life births after NC- or c-ICSI treatment in our hospital. 139/254 (55%) met the inclusion criteria of our study [NC-ICSI: 98/139 (71%), c-ICSI: 41/139 (29%)]. The drop-out rate within 24 months was 49% (68/139) (NC-ICSI: 53% (52/98), c-ICSI: 39% (16/41)).

### 3.2 Parental characteristics

No differences in maternal characteristics were found between the two groups concerning age, ethnicity, educational level, pre-pregnancy BMI, reason for infertility, age, or percentage of chronic disease (Table 1; Supplementary Table SI). Further there was no difference in parental height or weight (Table 2).

**Table 1:** Parental characteristics, stratified by stimulation scheme.

	NC-ICSI		c-ICSI		*P*-value*
	*N* = 98		*N* = 41		
	*n*	%	*n*	%	
Age of mother at conception (years)	34	3	34	5	0.575
Age of father at conception (years)	38	6	36	5	0.220
Parity					0.412
First	75	77	35	85	
Second or further	23	23	6	15	
Period of unfulfilled desire for children before treatment					0.782
Unknown	1	1	2	5	
1 year or less	17	17	6	15	
>1–2 years	18	18	8	20	
>2 years	62	63	25	61	
Smoking during pregnancy					0.644
Unknown	8	8	1	2	
No	87	89	39	95	
Yes	3	3	1	2	
BMI of mother					0.652
Underweight (BMI <18.5)	7	7	5	12	
Normal weight (BMI 18.5–24.9)	78	80	31	76	
Overweight (BMI 25–29.9)	12	12	3	7	
Obese (>30)	1	1	2	5	

NC-ICSI: parents undergoing natural cycle *in vitro* fertilization, c-ICSI: parents undergoing conventional *in vitro* fertilization, *p-values derived from Wilcoxon rank-sum (Mann–Whitney) tests, *p*-value less than 0.05 is considered statistically significant, BMI, body mass index.

**Table 2:** Parental height and weight, stratified by stimulation scheme.

	NC-ICSI					c-ICSI					*p*-value*
	*N* = 98					*N* = 41					
	*n* avail	% mis	Median	P5	P95	*n* avail	% mis	Median	P5	P95	
Height of mother (cm)	98	0	167	158	178	98	0	165	160	180	0.582
Weight of mother (Kg)	98	0	60	48	77	41	0	58	48	80	0.415
Height of father (cm)	89	9	180	172	193	38	7	180	172	190	0.312
Weight of father (Kg)	98	0	80	64	96	41	0	80	65	110	0.929

NC-ICSI, Natural Cycle ICSI; c-ICSI, conventional ICSI; *p-values derived from Wilcoxon rank-sum (Mann–Whitney) tests, g: gram, cm: centimeter, SDS: standard deviation score.

### 3.3 Birth characteristics

Birth characteristics are summarized in Table 3. Both ART groups were similar as to the sex of the child and number of children born preterm, low birth weight, and small for gestational age (Supplementary Tables SII and SIII). One child in the NC-ICSI group had a ventricular septal defect and hypospadias. All others were born healthy. The admission rate to the neonatal care unit was four percent. The breastfeeding rate was high in both groups: 81/98 (82%) of the newborns in the NC-ICSI group and 30/41 (73%) in the c-ICSI were breastfed for longer than three months (Supplementary Table SII).

**Table 3:** Auxologic data of children born after NC-ICSI and c-ICSI, stratified by stimulation scheme.

Time point at measurement	NC-ICSI					c-ICSI					*p*-value*
	*N* = 98					*N* = 41					
	*n* avail	% mis	Median	P5	P95	*n* avail	% mis	Median	P5	P95	
Gestational age	98	0	39	37	41	41	0	40	37	41	0.536
At birth											
Weight (kg)	98	0	3.4	2.4	4.2	41	0	3.3	2.5	4.4	0.611
Weight (SDS)	98	0	0.1	−1.8	1.3	41	0	−0.3	−1.5	2.1	0.525
Length (cm)	98	0	50.0	45.0	54.0	40	2	50.0	45.5	53.5	0.475
Length (SDS)	98	0	−0.5	−2.3	0.5	40	2	−0.9	−2.6	0.7	0.379
Head circumference (cm)	92	6	35.0	32.0	37.0	39	5	34.0	32.0	37.5	0.335
Head circumference (SDS)	92	6	0.1	−1.8	1.3	39	5	−0.2	−1.8	1.7	0.521
BMI (kg/m^2^)	98	0	13.5	11.0	15.3	40	2	13.2	11.3	16.1	0.925
BMI (SDS)	98	0	0.1	−2.1	1.5	40	2	−0.2	−1.8	1.9	0.920
At 1 month											
Weight (kg)	92	6	4.2	3.1	5.1	41	0	4.3	3.1	5.3	0.946
Weight (SDS)	92	6	−0.1	−2.2	1.2	41	0	−0.1	−2.3	1.3	0.677
Length (cm)	91	7	54.5	49.0	58.0	39	5	54.5	49.6	58.0	0.840
Length (SDS)	91	7	0.2	−2.4	1.7	39	5	0.2	−2.6	1.9	0.576
Head circumference (cm)	90	8	37.1	34.5	39.2	40	2	37.5	35.2	39.0	0.826
Head circumference (SDS)	90	8	0.3	−1.9	1.8	40	2	0.6	−1.5	2.2	0.705
BMI (kg/m^2^)	90	8	14.3	12.2	16.7	39	5	14.4	11.5	16.5	0.898
BMI (SDS)	90	8	−0.4	−2.0	1.2	39	5	−0.1	−2.5	1.1	0.804
At 2 months											
Weight (kg)	84	14	5.3	4.0	6.3	40	2	5.3	4.2	6.4	0.740
Weight (SDS)	84	14	−0.1	−1.9	1.1	40	2	−0.1	−2.0	1.2	0.889
Length (cm)	86	12	58.0	52.0	62.0	41	0	58.0	54.1	61.0	0.895
Length (SDS)	86	12	0.2	−2.5	1.8	41	0	0.1	−1.9	1.4	0.963
Head circumference (cm)	86	12	39.0	36.5	41.0	39	5	39.1	37.0	41.0	0.771
Head circumference (SDS)	86	12	0.1	−1.7	1.5	39	5	0.6	−1.3	1.5	0.413
BMI (kg/m2)	84	14	15.7	13.9	17.6	40	2	15.4	13.4	18.4	0.748
BMI (SDS)	84	14	−0.2	−1.8	0.9	40	2	−0.5	−2.0	1.4	0.949
At 4 months											
Weight (kg)	74	24	6.5	5.0	8.0	35	15	6.4	5.4	8.2	0.869
Weight (SDS)	74	24	−0.2	−2.2	1.3	35	15	−0.2	−1.9	1.7	0.974
Length (cm)	73	26	63.0	58.0	68.0	35	15	63.7	59.5	68.0	0.400
Length (SDS)	73	26	0.1	−2.3	2.0	35	15	0.4	−1.9	2.0	0.207
Head circumference (cm)	74	24	41.2	39.2	43.8	34	17	41.1	39.0	44.0	0.910
Head circumference (SDS)	74	24	0.0	−1.7	1.7	34	17	0.1	−1.7	1.7	0.698
BMI (kg/m^2^)	72	27	16.5	13.8	19.0	34	17	16.0	13.6	19.7	0.448
BMI (SDS)	72	27	−0.3	−2.1	1.2	34	17	−0.6	−2.3	1.6	0.440
At 6 months											
Weight (kg)	69	30	7.6	5.9	9.1	33	20	7.4	6.1	9.6	0.261
Weight (SDS)	69	30	0.0	−1.8	1.2	33	20	−0.3	−1.6	2.2	0.311
Length (cm)	69	30	67.0	62.0	71.5	35	15	66.5	62.5	71.5	0.741
Length (SDS)	69	30	0.2	−1.9	1.9	35	15	0.2	−1.7	1.9	0.986
Head circumference (cm)	70	29	43.2	41.0	45.0	33	20	43.0	40.8	46.2	0.793
Head circumference (SDS)	70	29	0.0	−1.6	1.5	33	20	0.0	−1.7	2.3	0.807
BMI (kg/m^2^)	67	32	16.7	14.4	18.9	32	22	16.2	14.1	19.6	0.309
BMI (SDS)	67	32	−0.4	−1.8	1.1	32	22	−0.7	−2.2	1.6	0.268
At 12 months											
Weight (kg)	74	24	9.2	7.1	11.4	36	12	9.0	7.7	11.7	0.437
Weight (SDS)	74	24	0.0	−1.9	1.8	36	12	−0.1	−1.7	2.1	0.726
Length (cm)	74	24	75.0	70.0	80.0	35	15	74.5	71.0	81.0	0.956
Length (SDS)	74	24	0.1	−2.1	1.8	35	15	0.0	−1.6	2.2	0.728
Head circumference (cm)	73	26	46.0	44.0	48.0	33	20	46.0	44.0	48.6	0.932
Head circumference (SDS)	73	26	−0.1	−1.7	1.1	33	20	0.0	−1.7	1.6	0.618
BMI (kg/m^2^)	73	26	16.6	14.3	19.2	34	17	16.0	14.2	19.2	0.166
BMI (SDS)	73	26	0.0	−2.1	1.6	34	17	−0.3	−2.0	1.8	0.211
At 18 months											
Weight (kg)	66	33	10.9	8.2	13.2	35	15	10.8	9.0	13.4	0.753
Weight (SDS)	66	33	0.2	−1.9	1.9	35	15	0.3	−1.4	1.9	0.920
Length (cm)	64	35	82.0	77.9	88.0	33	20	82.0	77.0	87.5	0.967
Length (SDS)	64	35	0.2	−1.4	2.1	33	20	0.4	−1.6	1.9	0.681
Head circumference (cm)	64	35	48.0	45.3	50.0	32	22	47.5	45.2	50.5	0.683
Head circumference (SDS)	64	35	0.0	−2.5	1.2	32	22	−0.1	−1.7	1.7	0.855
BMI (kg/m^2^)	64	35	16.1	14.0	18.7	32	22	15.9	13.6	18.9	0.703
BMI (SDS)	64	35	0.1	−1.8	1.8	32	22	0.1	−2.0	1.9	0.816
At 24 months											
Weight (kg)	51	48	12.2	9.7	14.5	29	29	12.2	10.5	14.6	0.822
Weight (SDS)	51	48	0.3	−1.4	1.7	29	29	0.2	−1.2	1.8	0.822
Length (cm)	51	48	87.5	80.0	95.0	29	29	88.0	84.3	93.0	0.434
Length (SDS)	51	48	0.1	−2.0	2.5	29	29	0.4	−1.2	1.7	0.305
Head circumference (cm)	49	50	49.0	46.4	51.0	26	37	48.6	46.5	51.3	0.664
Head circumference (SDS)	49	50	−0.2	−2.1	1.2	26	37	0.2	−1.7	1.4	0.518
BMI (kg/m^2^)	50	49	15.7	13.8	18.7	29	29	15.7	14.3	18.3	0.871
BMI (SDS)	50	49	0.2	−1.8	2.1	29	29	0.0	−1.2	1.9	0.976

NC-ICSI, natural cycle ICSI; c-ICSI: conventional ICSI, *p-values derived from Wilcoxon rank-sum (Mann–Whitney) tests, *p*-value less than 0.05 is considered statistically significant, BMI, body mass index in (kg/m^2^); SDS, standard deviation score; Kg, kilogram; cm, centimeter.

### 3.4 Growth characteristics

**Figure 1:**
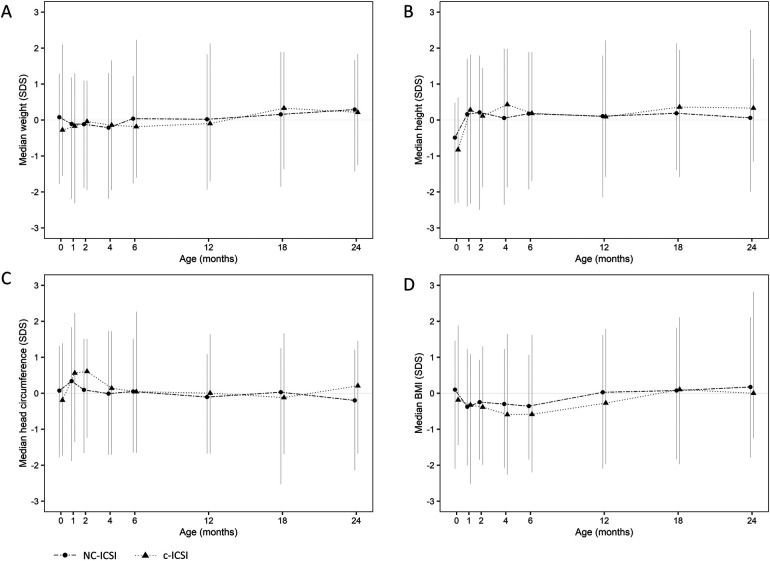
Standard deviation score (SDS) of weight **(A)**, height **(B)**, head circumference **(C)** and BMI **(D)** from 0 to 24 months of children born after c-ICSI and NC-ICSI. NC-ICSI, Natural Cycle ICSI; c-ICSI, conventional ICSI; SDS, standard deviation score; BMI, body mass index in (kg/m^2^).

On average, seven measurements were available for each child between delivery and the age of 24 months. ﻿Growth measurements at the different time points did not differ between the two groups in weight, height, head circumference or BMI (Figure 1 and Table 3). Both ART groups had slightly lower height (SDS) at birth compared with to Swiss national growth references. By the age of one month, they had caught up on length. When we excluded children born preterm or only very preterm children, we found similar results (Supplementary Tables SV and SVI). There was no difference in weight-, length-, head circumference or BMI gain within 0–2 months, 2–12 months as well as 12–24 months (Supplementary Table SIV).

## 4 Discussion

This is a cohort study about the height and weight gains of children conceived by ICSI with or without gonadotropin stimulation. Reassuringly, no difference in height or weight gain was observed when the two groups were compared or when they were compared to the Swiss national growth references.

The study has four strengths: first, complete and detailed information on patients (including parents), ART-treatment, and pregnancy outcomes with continuous growth data up to age of two years; second, the possibility to compare a remarkably high number of children born after NC-ICSI to children born following c-ICSI originating from one treatment site with standardized laboratory protocols for both groups; third, the complete inclusion of head circumference as an important parameter of health during childhood; and fourth, a valid comparison group including multiple growth parameters of 8,500 children by using the Swiss national growth references, *N* = 8500 (20). The use of SDS increases the prognostic value of our results.

Low sample size and a high drop-out rate are the limitations of the study. The size of the cohort follows the limited number of natural cycle treatments currently performed. The low response to follow-up might be associated with the costs for ART treatments in Switzerland which are not covered by the insurance. We postulate that parents who must pay the treatment themselves are less willing to take the extra effort to support research.

The drop-out rate of 49% within 24 months could lead to a selection bias towards healthier children which may conceal differences between the groups as well as differences between these two groups and the growth references.

Ceelen et al. were one of the first to sequentially report growth in the early childhood of ART children. Before that, most studies evaluated anthropometric parameters only at one follow-up timepoint, therefore the assessment of growth patterns was not possible. Ceelen et al. found lower weight, height, and BMI SDS up to the age of three months and differences disappeared within one year (27). In contrast to our study, their gonadotropin-stimulated group contained a high percentage of neonates born preterm (13% vs. 5% within our cohort). This is important to note because optimal growth velocity of preterm infants is faster, and therefore differs from term infants (28).

Both our study groups contained a high number of children with a birth length under the fifth percentile (19% in NC-ICSI and 15% in c-ICSI, *P* = 0.5). By the age of one month, they caught up and followed a normal growth pattern up to the age of two years. These results are in line with a recent registry study that includes 1,721 children born after ART (9). However, this is a large registry study including not only IVF/ICSI but also “unknown/other fertility treatments” and it is therefore not fully comparable to our study design.

### 4.1 Epigenetics and gonadotropins

Epigenetic modifications may contribute to an unfavorable perinatal outcome, especially lower birth weight, and an altered growth pattern. IVF/ICSI procedures happen in an extremely vulnerable timeframe, where large-scale reorganization of the epigenome takes place during which especially the ovarian hyperstimulation with gonadotropins used for stimulation has been assumed to interfere with epigenetic regulations (29, 30). A number of studies have found an influence of gonadotropins on perinatal outcome (11, 31). However, other studies have found no direct association between gonadotropin dosage and birth weight (10). This is consistent with our study observing no difference in weight and length gain between groups. The controversial results might be due to the different cohorts and stimulation protocols. The cohort of Mak et al. (31) consists of a very high percentage of children conceived of fathers with male factor infertility, and this study used a different protocol for c-IVF cycles. Pelinck et al. (11) compared c-IVF to a minimal stimulation IVF protocol and only mothers up to the age of 36 years (good-prognosis patients) were included in the study. This assumes that epigenetic modifications are multifactorial, and the single effect of gonadotropin stimulations is small.

### 4.2 Comparison groups and prognostic value of growth patterns

In general, it remains very difficult to compare various studies due to large differences regarding, e.g., selection of controls, treatment schedules, culture media, inclusion/exclusion of children and breastfeeding duration (32). Growth patterns are directly associated with nutritional trends, like breastfeeding duration. Longer duration of exclusive or partial breastfeeding is inversely associated with growth rates in infancy (33). Breastfeeding within our cohort was analyzed separately, showing no difference in breastfeeding initiation or duration after treatment cycles with or without gonadotropins (21).

Associations between intrauterine growth restriction and low birth weight with a higher prevalence of cardiometabolic diseases in later life have been demonstrated (34, 35). However, higher cardiovascular morbidity is potentially modified through postnatal growth patterns (7). A follow-up study from Ceelen et al. showed that early childhood growth (between age one to three years) was associated with a higher systolic blood pressure at follow-up (between age 8–18 years) which may be a predictor for cardiovascular disease in later life. Early catch-up growth during the first year of life of ART children was not associated with a higher blood pressure and is therefore assumed to be a physiological compensatory process to make up for low birth weight (27). However, we did not observe a catch-up growth in our cohort.

Growth, including height, weight and head circumference, is a good parameter to observe the general health of a child and might be a reasonable parameter to estimate long-term cardiometabolic risk. Longitudinal growth parameters are therefore very valuable. In our study we could not find an association of gonadotropin stimulation on childhood growth between birth and two years. These results are assuring and showing that gonadotropin stimulation in IVF/ICSI is a safe option. We promote further studies observing longitudinal growth parameters of children conceived after IVF/ICSI.

## 5 Conclusion

In our study, gonadotropin stimulation did not affect growth patterns in children conceived after NC-ICSI compared to c-ICSI. Further, in the first two years of life, children from the Bern IVF cohort did not show differences in auxologic characteristics compared to the Swiss national growth references. However, larger studies with a longer follow-up time are important to better identify factors associated with different growth patterns and their potential association with cardiovascular disease later in life.

## Data Availability

The original contributions presented in the study are included in the article/Supplementary Material, further inquiries can be directed to the corresponding author.
