# Supplementary material for: Childhood growth of singletons conceived following intracytoplasmic sperm injection – irrelevance of gonadotropin stimulation
Source: Front Reprod Health. 2024 Sep 23;6:1453697. doi: 10.3389/frph.2024.1453697 (PMC11464956; doi:10.3389/frph.2024.1453697)
Supplement: Supplementary file 1 [file Table1.docx]

**Table I: Parental characteristics, stratified by stimulation scheme**

|  | **NC-ICSI** |  | **c-ICSI** |  | **P-value*** |
| --- | --- | --- | --- | --- | --- |
|  | **N =98** |  | **N =41** |  |  |
|  | **n** | **%** | **n** | **%** |  |
| **Age of mother at conception** (years) | 34 | 3 | 34 | 5 | **0.575** |
| **Age of father at conception** (years) | 38 | 6 | 36 | 5 | **0.220** |
| **Parity** |  |  |  |  | **0.412** |
| First | 75 | 77 | 35 | 85 |  |
| Second or further | 23 | 23 | 6 | 15 |  |
| **Period of unfulfilled desire for children before treatment** |  |  |  |  | **0.782** |
| Unknown | 1 | 1 | 2 | 5 |  |
| 1 year or less | 17 | 17 | 6 | 15 |  |
| >1 to 2 years | 18 | 18 | 8 | 20 |  |
| >2 years | 62 | 63 | 25 | 61 |  |
| **Smoking during pregnancy** |  |  |  |  | **0.644** |
| Unknown | 8 | 8 | 1 | 2 |  |
| No | 87 | 89 | 39 | 95 |  |
| Yes | 3 | 3 | 1 | 2 |  |
| **BMI of mother** |  |  |  |  | **0.652** |
| Underweight ( BMI <18.5) | 7 | 7 | 5 | 12 |  |
| Normal weight (BMI 18.5-24.9) | 78 | 80 | 31 | 76 |  |
| Overweight (BMI 25 – 29.9) | 12 | 12 | 3 | 7 |  |
| Obese (>30 | 1 | 1 | 2 | 5 |  |

NC-ICSI: Parents undergoing natural cycle in-vitro fertilization , c-ICSI: Parents undergoing conventional in-vitro fertilization, *p-values derived from Wilcoxon rank-sum (Mann–Whitney) tests, p-value less than 0.05 is considered statistically significant, BMI: body mass index
