# Supplementary material for: Childhood growth of singletons conceived following intracytoplasmic sperm injection – irrelevance of gonadotropin stimulation
Source: Front Reprod Health. 2024 Sep 23;6:1453697. doi: 10.3389/frph.2024.1453697 (PMC11464956; doi:10.3389/frph.2024.1453697)
Supplement: Supplementary file 2 [file Table2.docx]

**Table II: Parental Height and Weight, stratified by stimulation scheme**

|  | **NC-ICSI** | | | | | **c-ICSI** | | | | | **p-value*** |
| --- | --- | --- | --- | --- | --- | --- | --- | --- | --- | --- | --- |
|  | **N=98** | | | | | **N=41** | | | | |  |
|  | **n avail** | **% mis** | **Median** | **P5** | **P95** | **n avail** | **% mis** | **Median** | **P5** | **P95** |  |
|  |  |  |  |  |  |  |  |  |  |  |  |
| **Height of mother (cm)** | **98** | **0** | **167** | **158** | **178** | **98** | **0** | **165** | **160** | **180** | **0.582** |
| **Weight of mother (Kg)** | 98 | 0 | 60 | 48 | 77 | 41 | 0 | 58 | 48 | 80 | 0.415 |
| **Height of father (cm)** | 89 | 9 | 180 | 172 | 193 | 38 | 7 | 180 | 172 | 190 | 0.312 |
| **Weight of father (Kg)** | 98 | 0 | 80 | 64 | 96 | 41 | 0 | 80 | 65 | 110 | 0.929 |

NC-ICSI: Parents undergoing natural cycle in-vitro fertilization , c-ICSI: Parents undergoing conventional in-vitro fertilization, *p-values derived from Wilcoxon rank-sum (Mann–Whitney) tests, p-value less than 0.05 is considered as statistically significant.
