# Supplementary material for: Childhood growth of singletons conceived following intracytoplasmic sperm injection – irrelevance of gonadotropin stimulation
Source: Front Reprod Health. 2024 Sep 23;6:1453697. doi: 10.3389/frph.2024.1453697 (PMC11464956; doi:10.3389/frph.2024.1453697)
Supplement: Supplementary file 4 [file Table4.docx]

**Supplementary Table SIV: Auxologic data of IVF-children: means and SDSs from the measurements up to the age of 24 months and the growth velocity between 0-2, 2-12 as well as 12-24 months**

| **Time point at measurement** | **NC-ICSI** | | | | | **c-ICSI** | | | | | **p-value*** |
| --- | --- | --- | --- | --- | --- | --- | --- | --- | --- | --- | --- |
|  | **N=98** | | | | | **N=41** | | | | |  |
|  | **n avail** | **% mis** | **Median** | **P5** | **P95** | **n avail** | **% mis** | **Median** | **P5** | **P95** |  |
|  |  |  |  |  |  |  |  |  |  |  |  |
| **At birth** |  |  |  |  |  |  |  |  |  |  |  |
| **Weight (kg)** | 98 | 0 | 3.4 | 2.4 | 4.2 | 41 | 0 | 3.3 | 2.5 | 4.4 | 0.611 |
| **Weight (SDS)** | 98 | 0 | 0.1 | -1.8 | 1.3 | 41 | 0 | -0.3 | -1.5 | 2.1 | 0.525 |
| **Length (cm)** | 98 | 0 | 50.0 | 45.0 | 54.0 | 40 | 2 | 50.0 | 45.5 | 53.5 | 0.475 |
| **Length (SDS)** | 98 | 0 | -0.5 | -2.3 | 0.5 | 40 | 2 | -0.9 | -2.6 | 0.7 | 0.379 |
| **Head circumference (cm)** | 92 | 6 | 35.0 | 32.0 | 37.0 | 39 | 5 | 34.0 | 32.0 | 37.5 | 0.335 |
| **Head circumference (SDS)** | 92 | 6 | 0.1 | -1.8 | 1.3 | 39 | 5 | -0.2 | -1.8 | 1.7 | 0.521 |
| **BMI (kg/m2)** | 98 | 0 | 13.5 | 11.0 | 15.3 | 40 | 2 | 13.2 | 11.3 | 16.1 | 0.925 |
| **BMI (SDS)** | 98 | 0 | 0.1 | -2.1 | 1.5 | 40 | 2 | -0.2 | -1.8 | 1.9 | 0.920 |
|  |  |  |  |  |  |  |  |  |  |  |  |
| **At 1 month** |  |  |  |  |  |  |  |  |  |  |  |
| **Weight (kg)** | 92 | 6 | 4.2 | 3.1 | 5.1 | 41 | 0 | 4.3 | 3.1 | 5.3 | 0.946 |
| **Weight (SDS)** | 92 | 6 | -0.1 | -2.2 | 1.2 | 41 | 0 | -0.1 | -2.3 | 1.3 | 0.677 |
| **Length (cm)** | 91 | 7 | 54.5 | 49.0 | 58.0 | 39 | 5 | 54.5 | 49.6 | 58.0 | 0.840 |
| **Length (SDS)** | 91 | 7 | 0.2 | -2.4 | 1.7 | 39 | 5 | 0.2 | -2.6 | 1.9 | 0.576 |
| **Head circumference (cm)** | 90 | 8 | 37.1 | 34.5 | 39.2 | 40 | 2 | 37.5 | 35.2 | 39.0 | 0.826 |
| **Head circumference (SDS)** | 90 | 8 | 0.3 | -1.9 | 1.8 | 40 | 2 | 0.6 | -1.5 | 2.2 | 0.705 |
| **BMI (kg/m2)** | 90 | 8 | 14.3 | 12.2 | 16.7 | 39 | 5 | 14.4 | 11.5 | 16.5 | 0.898 |
| **BMI (SDS)** | 90 | 8 | -0.4 | -2.0 | 1.2 | 39 | 5 | -0.1 | -2.5 | 1.1 | 0.804 |
|  |  |  |  |  |  |  |  |  |  |  |  |
| **At 2 months** |  |  |  |  |  |  |  |  |  |  |  |
| **Weight (kg)** | 84 | 14 | 5.3 | 4.0 | 6.3 | 40 | 2 | 5.3 | 4.2 | 6.4 | 0.740 |
| **Weight (SDS)** | 84 | 14 | -0.1 | -1.9 | 1.1 | 40 | 2 | -0.1 | -2.0 | 1.2 | 0.889 |
| **Length (cm)** | 86 | 12 | 58.0 | 52.0 | 62.0 | 41 | 0 | 58.0 | 54.1 | 61.0 | 0.895 |
| **Length (SDS)** | 86 | 12 | 0.2 | -2.5 | 1.8 | 41 | 0 | 0.1 | -1.9 | 1.4 | 0.963 |
| **Head circumference (cm)** | 86 | 12 | 39.0 | 36.5 | 41.0 | 39 | 5 | 39.1 | 37.0 | 41.0 | 0.771 |
| **Head circumference (SDS)** | 86 | 12 | 0.1 | -1.7 | 1.5 | 39 | 5 | 0.6 | -1.3 | 1.5 | 0.413 |
| **BMI (kg/m2)** | 84 | 14 | 15.7 | 13.9 | 17.6 | 40 | 2 | 15.4 | 13.4 | 18.4 | 0.748 |
| **BMI (SDS)** | 84 | 14 | -0.2 | -1.8 | 0.9 | 40 | 2 | -0.5 | -2.0 | 1.4 | 0.949 |
|  |  |  |  |  |  |  |  |  |  |  |  |
| **At 4 months** |  |  |  |  |  |  |  |  |  |  |  |
| **Weight (kg)** | 74 | 24 | 6.5 | 5.0 | 8.0 | 35 | 15 | 6.4 | 5.4 | 8.2 | 0.869 |
| **Weight (SDS)** | 74 | 24 | -0.2 | -2.2 | 1.3 | 35 | 15 | -0.2 | -1.9 | 1.7 | 0.974 |
| **Length (cm)** | 73 | 26 | 63.0 | 58.0 | 68.0 | 35 | 15 | 63.7 | 59.5 | 68.0 | 0.400 |
| **Length (SDS)** | 73 | 26 | 0.1 | -2.3 | 2.0 | 35 | 15 | 0.4 | -1.9 | 2.0 | 0.207 |
| **Head circumference (cm)** | 74 | 24 | 41.2 | 39.2 | 43.8 | 34 | 17 | 41.1 | 39.0 | 44.0 | 0.910 |
| **Head circumference (SDS)** | 74 | 24 | 0.0 | -1.7 | 1.7 | 34 | 17 | 0.1 | -1.7 | 1.7 | 0.698 |
| **BMI (kg/m2)** | 72 | 27 | 16.5 | 13.8 | 19.0 | 34 | 17 | 16.0 | 13.6 | 19.7 | 0.448 |
| **BMI (SDS)** | 72 | 27 | -0.3 | -2.1 | 1.2 | 34 | 17 | -0.6 | -2.3 | 1.6 | 0.440 |
|  |  |  |  |  |  |  |  |  |  |  |  |
| **At 6 months** |  |  |  |  |  |  |  |  |  |  |  |
| **Weight (kg)** | 69 | 30 | 7.6 | 5.9 | 9.1 | 33 | 20 | 7.4 | 6.1 | 9.6 | 0.261 |
| **Weight (SDS)** | 69 | 30 | 0.0 | -1.8 | 1.2 | 33 | 20 | -0.3 | -1.6 | 2.2 | 0.311 |
| **Length (cm)** | 69 | 30 | 67.0 | 62.0 | 71.5 | 35 | 15 | 66.5 | 62.5 | 71.5 | 0.741 |
| **Length (SDS)** | 69 | 30 | 0.2 | -1.9 | 1.9 | 35 | 15 | 0.2 | -1.7 | 1.9 | 0.986 |
| **Head circumference (cm)** | 70 | 29 | 43.2 | 41.0 | 45.0 | 33 | 20 | 43.0 | 40.8 | 46.2 | 0.793 |
| **Head circumference (SDS)** | 70 | 29 | 0.0 | -1.6 | 1.5 | 33 | 20 | 0.0 | -1.7 | 2.3 | 0.807 |
| **BMI (kg/m2)** | 67 | 32 | 16.7 | 14.4 | 18.9 | 32 | 22 | 16.2 | 14.1 | 19.6 | 0.309 |
| **BMI (SDS)** | 67 | 32 | -0.4 | -1.8 | 1.1 | 32 | 22 | -0.7 | -2.2 | 1.6 | 0.268 |
|  |  |  |  |  |  |  |  |  |  |  |  |
| **At 12 months** |  |  |  |  |  |  |  |  |  |  |  |
| **Weight (kg)** | 74 | 24 | 9.2 | 7.1 | 11.4 | 36 | 12 | 9.0 | 7.7 | 11.7 | 0.437 |
| **Weight (SDS)** | 74 | 24 | 0.0 | -1.9 | 1.8 | 36 | 12 | -0.1 | -1.7 | 2.1 | 0.726 |
| **Length (cm)** | 74 | 24 | 75.0 | 70.0 | 80.0 | 35 | 15 | 74.5 | 71.0 | 81.0 | 0.956 |
| **Length (SDS)** | 74 | 24 | 0.1 | -2.1 | 1.8 | 35 | 15 | 0.0 | -1.6 | 2.2 | 0.728 |
| **Head circumference (cm)** | 73 | 26 | 46.0 | 44.0 | 48.0 | 33 | 20 | 46.0 | 44.0 | 48.6 | 0.932 |
| **Head circumference (SDS)** | 73 | 26 | -0.1 | -1.7 | 1.1 | 33 | 20 | 0.0 | -1.7 | 1.6 | 0.618 |
| **BMI (kg/m2)** | 73 | 26 | 16.6 | 14.3 | 19.2 | 34 | 17 | 16.0 | 14.2 | 19.2 | 0.166 |
| **BMI (SDS)** | 73 | 26 | 0.0 | -2.1 | 1.6 | 34 | 17 | -0.3 | -2.0 | 1.8 | 0.211 |
|  |  |  |  |  |  |  |  |  |  |  |  |
| **At 18 months** |  |  |  |  |  |  |  |  |  |  |  |
| **Weight (kg)** | 66 | 33 | 10.9 | 8.2 | 13.2 | 35 | 15 | 10.8 | 9.0 | 13.4 | 0.753 |
| **Weight (SDS)** | 66 | 33 | 0.2 | -1.9 | 1.9 | 35 | 15 | 0.3 | -1.4 | 1.9 | 0.920 |
| **Length (cm)** | 64 | 35 | 82.0 | 77.9 | 88.0 | 33 | 20 | 82.0 | 77.0 | 87.5 | 0.967 |
| **Length (SDS)** | 64 | 35 | 0.2 | -1.4 | 2.1 | 33 | 20 | 0.4 | -1.6 | 1.9 | 0.681 |
| **Head circumference (cm)** | 64 | 35 | 48.0 | 45.3 | 50.0 | 32 | 22 | 47.5 | 45.2 | 50.5 | 0.683 |
| **Head circumference (SDS)** | 64 | 35 | 0.0 | -2.5 | 1.2 | 32 | 22 | -0.1 | -1.7 | 1.7 | 0.855 |
| **BMI (kg/m2)** | 64 | 35 | 16.1 | 14.0 | 18.7 | 32 | 22 | 15.9 | 13.6 | 18.9 | 0.703 |
| **BMI (SDS)** | 64 | 35 | 0.1 | -1.8 | 1.8 | 32 | 22 | 0.1 | -2.0 | 1.9 | 0.816 |
|  |  |  |  |  |  |  |  |  |  |  |  |
| **At 24 months** |  |  |  |  |  |  |  |  |  |  |  |
| **Weight (kg)** | 51 | 48 | 12.2 | 9.7 | 14.5 | 29 | 29 | 12.2 | 10.5 | 14.6 | 0.822 |
| **Weight (SDS)** | 51 | 48 | 0.3 | -1.4 | 1.7 | 29 | 29 | 0.2 | -1.2 | 1.8 | 0.822 |
| **Length (cm)** | 51 | 48 | 87.5 | 80.0 | 95.0 | 29 | 29 | 88.0 | 84.3 | 93.0 | 0.434 |
| **Length (SDS)** | 51 | 48 | 0.1 | -2.0 | 2.5 | 29 | 29 | 0.4 | -1.2 | 1.7 | 0.305 |
| **Head circumference (cm)** | 49 | 50 | 49.0 | 46.4 | 51.0 | 26 | 37 | 48.6 | 46.5 | 51.3 | 0.664 |
| **Head circumference (SDS)** | 49 | 50 | -0.2 | -2.1 | 1.2 | 26 | 37 | 0.2 | -1.7 | 1.4 | 0.518 |
| **BMI (kg/m2)** | 50 | 49 | 15.7 | 13.8 | 18.7 | 29 | 29 | 15.7 | 14.3 | 18.3 | 0.871 |
| **BMI (SDS)** | 50 | 49 | 0.2 | -1.8 | 2.1 | 29 | 29 | 0.0 | -1.2 | 1.9 | 0.976 |
|  |  |  |  |  |  |  |  |  |  |  |  |
| **Weight gain (SDS)** |  |  |  |  |  |  |  |  |  |  |  |
| **Delta 0-2 months** | 84 | 14 | -0.2 | -1.7 | 1.1 | 40 | 2 | -0.1 | -1.7 | 1.4 | 0.549 |
| **Delta 2-12 months** | 66 | 33 | 0.3 | -0.8 | 2.1 | 35 | 15 | 0.4 | -1.0 | 1.7 | 0.484 |
| **Delta 12-24 months** | 49 | 50 | 0.2 | -0.6 | 1.0 | 28 | 32 | 0.4 | -0.2 | 1.0 | 0.294 |
|  |  |  |  |  |  |  |  |  |  |  |  |
| **Length gain (SDS)** |  |  |  |  |  |  |  |  |  |  |  |
| **Delta 0-2 months** | 86 | 12 | 0.9 | -1.4 | 2.3 | 40 | 2 | 1.0 | -1.2 | 1.9 | 0.307 |
| **Delta 2-12 months** | 68 | 31 | 0.2 | -0.9 | 1.6 | 35 | 15 | 0.2 | -1.6 | 2.0 | 0.903 |
| **Delta 12-24 months** | 49 | 50 | -0.1 | -0.7 | 1.0 | 27 | 34 | 0.1 | -1.1 | 1.5 | 0.672 |
|  |  |  |  |  |  |  |  |  |  |  |  |
| **Head circumference gain (SDS)** |  |  |  |  |  |  |  |  |  |  |  |
| **Delta 0-2 months** | 80 | 18 | 0.2 | -1.6 | 2.2 | 37 | 10 | 0.3 | -1.0 | 2.2 | 0.267 |
| **Delta 2-12 months** | 67 | 32 | -0.2 | -1.5 | 0.9 | 31 | 24 | -0.4 | -1.7 | 1.0 | 0.852 |
| **Delta 12-24 months** | 47 | 52 | 0.1 | -0.9 | 0.9 | 23 | 44 | 0.1 | -0.6 | 0.7 | 0.920 |
|  |  |  |  |  |  |  |  |  |  |  |  |
| **BMI gain (SDS)** |  |  |  |  |  |  |  |  |  |  |  |
| **Delta 0-2 months** | 84 | 14 | -0.4 | -1.8 | 1.6 | 39 | 5 | -0.7 | -2.4 | 2.2 | 0.913 |
| **Delta 2-12 months** | 65 | 34 | 0.4 | -0.9 | 2.0 | 33 | 20 | -0.1 | -1.3 | 1.6 | 0.126 |
| **Delta 12-24 months** | 47 | 52 | 0.5 | -1.1 | 1.4 | 26 | 37 | 0.7 | -0.4 | 1.2 | 0.440 |

NC-ICSI: Natural Cycle ICSI, c-ICSI: conventional ICSI, *p-values derived from Wilcoxon rank-sum (Mann–Whitney) tests , BMI: body mass index in (kg/m2), SDS: Standard deviation score, Kg: kilogram, cm: centimeter
