# Supplementary material for: Childhood growth of singletons conceived following intracytoplasmic sperm injection – irrelevance of gonadotropin stimulation
Source: Front Reprod Health. 2024 Sep 23;6:1453697. doi: 10.3389/frph.2024.1453697 (PMC11464956; doi:10.3389/frph.2024.1453697)
Supplement: Supplementary file 5 [file Table5.docx]

**Supplementary Table SV: Auxologic data of IVF-children: means and SDSs^b^ from the**

**measurements up to the age of 24 months and the growth velocity between 0-2, 2-12 as well as 12-24 months with the exclusion of preterms (≤ 36+6  weeks)**

| **Timepoint at measurement** | **NC-ICSI** | | | | | **c-ICSI** | | | | | | | | **p-value*** |
| --- | --- | --- | --- | --- | --- | --- | --- | --- | --- | --- | --- | --- | --- | --- |
|  | **N=92** | | | | | **N=38** | | | | | | | |  |
|  | **n avail** | **% mis** | **Median** | **P5** | **P95** | **n avail** | **% mis** | **Median** | | **P55** | | **P95** | |  |
| **At birth** |  |  |  |  |  |  |  |  | |  | |  | |  |
| **Weight (SDS)** | 92 | 0 | 0.1 | -1.4 | 1.3 | 38 | 0 | -0.1 | | -1.5 | | 2.4 | | 0.551 |
| **Length (SDS)** | 92 | 0 | -0.5 | -2.1 | 0.5 | 37 | 3 | -0.8 | | -2.3 | | 0.8 | | 0.430 |
| **Head circumference (SDS)** | 86 | 7 | 0.1 | -1.7 | 1.3 | 36 | 5 | -0.1 | | -1.7 | | 1.7 | | 0.376 |
| **BMI (SDS)** | 92 | 0 | 0.2 | -1.5 | 1.5 | 37 | 3 | 0.0 | | -2.1 | | 1.9 | | 0.870 |
|  |  |  |  |  |  |  |  |  | |  | |  | |  |
| **At 1 month** |  |  |  |  |  |  |  |  | |  | |  | |  |
| **Weight (SDS)** | 87 | 5 | -0.1 | -1.9 | 1.2 | 38 | 0 | 0.1 | -2.7 | | 1.7 | | 0.525 | |
| **Length (SDS)** | 86 | 7 | 0.2 | -2.2 | 1.7 | 36 | 5 | 0.4 | -2.6 | | 1.9 | | 0.507 | |
| **Head circumference (SDS)** | 85 | 8 | 0.6 | -1.4 | 1.8 | 37 | 3 | 0.6 | -0.9 | | 2.2 | | 0.709 | |
| **BMI (SDS)** | 85 | 8 | -0.3 | -1.8 | 1.2 | 36 | 5 | -0.1 | -2.5 | | 1.1 | | 0.694 | |
|  |  |  |  |  |  |  |  |  |  | |  | |  | |
| **At 2 months** |  |  |  |  |  |  |  |  |  | |  | |  | |
| **Weight (SDS)** | 80 | 13 | -0.1 | -1.8 | 1.2 | 37 | 3 | 0.1 | -2.1 | | 1.3 | | 0.727 | |
| **Length (SDS)** | 81 | 12 | 0.2 | -2.2 | 1.8 | 38 | 0 | 0.2 | -2.2 | | 1.8 | | 0.964 | |
| **Head circumference (SDS)** | 81 | 12 | 0.2 | -1.1 | 1.5 | 36 | 5 | 0.6 | -1.1 | | 1.5 | | 0.458 | |
| **BMI (SDS)** | 80 | 13 | -0.2 | -1.8 | 1.0 | 37 | 3 | -0.2 | -2.0 | | 1.4 | | 0.874 | |
|  |  |  |  |  |  |  |  |  |  | |  | |  | |
| **Between 3-5 months** |  |  |  |  |  |  |  |  |  | |  | |  | |
| **Weight (SDS)** | 76 | 17 | -0.2 | -1.8 | 1.3 | 33 | 13 | -0.1 | -1.9 | | 1.7 | | 0.807 | |
| **Length (SDS)** | 76 | 17 | 0.1 | -2.1 | 2.0 | 33 | 13 | 0.5 | -1.6 | | 2.0 | | 0.175 | |
| **Head circumference (SDS)** | 76 | 17 | 0.0 | -1.6 | 1.7 | 32 | 16 | 0.1 | -1.7 | | 1.7 | | 0.644 | |
| **BMI (SDS)** | 75 | 18 | -0.2 | -2.0 | 1.2 | 32 | 16 | -0.5 | -2.3 | | 1.6 | | 0.514 | |
|  |  |  |  |  |  |  |  |  |  | |  | |  | |
| **Between 6-10 months** |  |  |  |  |  |  |  |  |  | |  | |  | |
| **Weight (SDS)** | 65 | 29 | 0.0 | -1.6 | 1.2 | 30 | 21 | -0.1 | -1.6 | | 2.2 | | 0.387 | |
| **Length (SDS)** | 64 | 30 | 0.2 | -1.6 | 1.9 | 32 | 16 | 0.2 | -1.5 | | 1.9 | | 0.901 | |
| **Head circumference (SDS)** | 65 | 29 | 0.2 | -1.4 | 1.5 | 30 | 21 | 0.0 | -1.7 | | 2.3 | | 0.662 | |
| **BMI (SDS)** | 63 | 32 | -0.3 | -1.6 | 1.1 | 29 | 24 | -0.6 | -2.0 | | 1.6 | | 0.280 | |
|  |  |  |  |  |  |  |  |  |  | |  | |  | |
| **Between 11-15 months** |  |  |  |  |  |  |  |  |  | |  | |  | |
| **Weight (SDS)** | 79 | 14 | 0.0 | -1.5 | 1.8 | 35 | 8 | -0.1 | -1.7 | | 2.1 | | 0.531 | |
| **Length (SDS)** | 80 | 13 | 0.1 | -1.7 | 2.0 | 35 | 8 | 0.2 | -1.2 | | 2.2 | | 0.947 | |
| **Head circumference (SDS)** | 80 | 13 | 0.0 | -1.6 | 1.5 | 34 | 11 | 0.0 | -1.7 | | 1.6 | | 0.835 | |
| **BMI (SDS)** | 79 | 14 | 0.1 | -1.5 | 1.5 | 34 | 11 | -0.3 | -2.0 | | 1.8 | | 0.068 | |
|  |  |  |  |  |  |  |  |  |  | |  | |  | |
| **Between 16-20 months** |  |  |  |  |  |  |  |  |  | |  | |  | |
| **Weight (SDS)** | 73 | 21 | 0.2 | -1.0 | 2.0 | 35 | 8 | 0.4 | -1.4 | | 2.0 | | 0.748 | |
| **Length (SDS)** | 72 | 22 | 0.3 | -1.3 | 2.3 | 33 | 13 | 0.4 | -1.3 | | 1.9 | | 0.666 | |
| **Head circumference (SDS)** | 71 | 23 | 0.2 | -1.7 | 1.2 | 33 | 13 | -0.1 | -1.7 | | 1.7 | | 0.627 | |
| **BMI (SDS)** | 72 | 22 | 0.1 | -1.6 | 1.8 | 32 | 16 | 0.2 | -2.0 | | 2.1 | | 0.927 | |
|  |  |  |  |  |  |  |  |  |  | |  | |  | |
| **Between 21-25 months** |  |  |  |  |  |  |  |  |  | |  | |  | |
| **Weight (SDS)** | 54 | 41 | 0.3 | -1.4 | 1.7 | 28 | 26 | 0.4 | -1.2 | | 1.8 | | 0.688 | |
| **Length (SDS)** | 53 | 42 | 0.3 | -1.4 | 2.5 | 28 | 26 | 0.4 | -1.2 | | 1.9 | | 0.541 | |
| **Head circumference (SDS)** | 52 | 43 | 0.0 | -1.5 | 1.5 | 25 | 34 | 0.2 | -1.5 | | 1.4 | | 0.486 | |
| **BMI (SDS)** | 53 | 42 | 0.2 | -1.5 | 2.1 | 28 | 26 | 0.0 | -1.2 | | 1.9 | | 0.850 | |
|  |  |  |  |  |  |  |  |  |  | |  | |  | |
| **Weight gain (SDS)** |  |  |  |  |  |  |  |  |  | |  | |  | |
| **Delta 0-2 months** | 80 | 13 | -0.2 | -1.6 | 1.1 | 37 | 3 | -0.2 | -2.0 | | 1.7 | | 0.725 | |
| **Delta 2-12 months** | 71 | 23 | 0.3 | -0.9 | 1.6 | 34 | 11 | 0.2 | -1.0 | | 1.7 | | 0.247 | |
| **Delta 12-24 months** | 52 | 43 | 0.2 | -0.6 | 1.0 | 27 | 29 | 0.4 | -0.2 | | 1.0 | | 0.219 | |
|  |  |  |  |  |  |  |  |  |  | |  | |  | |
| **Length gain (SDS)** |  |  |  |  |  |  |  |  |  | |  | |  | |
| **Delta 0-2 months** | 81 | 12 | 0.9 | -1.0 | 2.3 | 37 | 3 | 1.0 | -1.2 | | 2.0 | | 0.535 | |
| **Delta 2-12 months** | 73 | 21 | 0.2 | -1.2 | 1.5 | 35 | 8 | 0.6 | -1.7 | | 2.0 | | 0.613 | |
| **Delta 12-24 months** | 52 | 43 | 0.0 | -0.7 | 1.4 | 27 | 29 | 0.1 | -1.1 | | 1.4 | | 0.897 | |
|  |  |  |  |  |  |  |  |  |  | |  | |  | |
| **Head circumference gain (SDS)** |  |  |  |  |  |  |  |  |  | |  | |  | |
| **Delta 0-2 months** | 75 | 18 | 0.3 | -1.5 | 2.2 | 34 | 11 | 0.4 | -1.0 | | 2.2 | | 0.268 | |
| **Delta 2-12 months** | 73 | 21 | -0.2 | -1.6 | 1.1 | 32 | 16 | -0.4 | -1.7 | | 1.0 | | 0.549 | |
| **Delta 12-24 months** | 51 | 45 | 0.0 | -0.9 | 0.9 | 24 | 37 | 0.1 | -1.0 | | 0.7 | | 0.654 | |
|  |  |  |  |  |  |  |  |  |  | |  | |  | |
| **BMI gain (SDS)** |  |  |  |  |  |  |  |  |  | |  | |  | |
| **Delta 0-2 months** | 80 | 13 | -0.5 | -1.9 | 1.4 | 36 | 5 | -0.7 | -2.4 | | 2.2 | | 0.698 | |
| **Delta 2-12 months** | 71 | 23 | 0.3 | -1.1 | 2.0 | 33 | 13 | -0.1 | -1.3 | | 1.5 | | 0.054 | |
| **Delta 12-24 months** | 51 | 45 | 0.5 | -1.1 | 1.4 | 26 | 32 | 0.7 | -0.4 | | 1.3 | | 0.132 | |

NC-ICSI: Natural Cycle ICSI, c-ICSI: conventional ICSI, *p-values derived from Wilcoxon rank-sum (Mann–Whitney) tests, BMI: body mass index in (kg/m2), SDS: Standard deviation score, Kg: kilogram, cm: centimeter
