# Supplementary material for: Childhood growth of singletons conceived following intracytoplasmic sperm injection – irrelevance of gonadotropin stimulation
Source: Front Reprod Health. 2024 Sep 23;6:1453697. doi: 10.3389/frph.2024.1453697 (PMC11464956; doi:10.3389/frph.2024.1453697)
Supplement: Supplementary file 6 [file Table6.docx]

**Supplementary Table SVI: Auxologic data of IVF-children: means and SDSs^b^ from the measurements up to the age of 24 months and the growth velocity between 0-2, 2-12 as well as 12-24 months with the exclusion of preterms (≤ 36+6  weeks)**

| **Timepoint of measurement** | **NC-ICSI** | | | |  | **C-ICSI** | | | | | **p-value*** |
| --- | --- | --- | --- | --- | --- | --- | --- | --- | --- | --- | --- |
|  | **N=88** | | | |  | **N=36** | | | | |  |
|  | **n avail** | **% mis** | **Median** | **P5** | **P95** | **n avail** | **% mis** | **Median** | **P5** | **P95** |  |
| **At birth** |  |  |  |  |  |  |  |  |  |  |  |
| **Weight (SDS)** | 88 | 0 | 0.1 | -1.3 | 1.3 | 36 | 0 | -0.1 | -1.5 | 2.4 | 0.642 |
| **Length (SDS)** | 88 | 0 | -0.5 | -2.1 | 0.5 | 35 | 3 | -0.8 | -2.3 | 0.8 | 0.371 |
| **Head circumference (SDS)** | 82 | 7 | 0.1 | -1.7 | 1.3 | 34 | 6 | -0.2 | -1.7 | 1.7 | 0.327 |
| **BMI**^a^ **(SDS)** | 88 | 0 | 0.3 | -1.3 | 1.5 | 35 | 3 | 0.0 | -1.4 | 1.9 | 0.966 |
|  |  |  |  |  |  |  |  |  |  |  |  |
| **At 1 month** |  |  |  |  |  |  |  |  |  |  |  |
| **Weight (SDS)** | 83 | 6 | 0.0 | -1.6 | 1.2 | 36 | 0 | 0.2 | -1.6 | 1.7 | 0.420 |
| **Length (SDS)** | 82 | 7 | 0.2 | -1.9 | 1.7 | 34 | 6 | 0.4 | -1.9 | 1.9 | 0.380 |
| **Head circumference (SDS)** | 81 | 8 | 0.6 | -1.4 | 1.8 | 35 | 3 | 0.6 | -0.9 | 2.2 | 0.700 |
| **BMI**^a^ **(SDS)** | 81 | 8 | -0.2 | -1.6 | 1.2 | 34 | 6 | 0.0 | -1.3 | 1.1 | 0.562 |
|  |  |  |  |  |  |  |  |  |  |  |  |
| **At 2 months** |  |  |  |  |  |  |  |  |  |  |  |
| **Weight (SDS)** | 77 | 13 | -0.1 | -1.8 | 1.2 | 35 | 3 | 0.2 | -1.8 | 1.3 | 0.581 |
| **Length (SDS)** | 78 | 11 | 0.3 | -2.2 | 1.8 | 36 | 0 | 0.2 | -2.2 | 1.8 | 0.879 |
| **Head circumference (SDS)** | 78 | 11 | 0.2 | -1.1 | 1.8 | 34 | 6 | 0.6 | -1.1 | 1.5 | 0.375 |
| **BMI** ^a^ **(SDS)** | 77 | 13 | -0.2 | -1.8 | 1.0 | 35 | 3 | 0.0 | -2.0 | 1.4 | 0.654 |
|  |  |  |  |  |  |  |  |  |  |  |  |
| **Between 3-5 months** |  |  |  |  |  |  |  |  |  |  |  |
| **Weight (SDS)** | 73 | 17 | -0.2 | -1.8 | 1.3 | 31 | 14 | 0.0 | -1.4 | 1.7 | 0.551 |
| **Length (SDS)** | 73 | 17 | 0.2 | -1.9 | 2.0 | 31 | 14 | 0.7 | -1.4 | 2.0 | 0.110 |
| **Head circumference (SDS)** | 73 | 17 | 0.0 | -1.5 | 1.7 | 30 | 17 | 0.1 | -1.7 | 1.7 | 0.495 |
| **BMI**^a^ **(SDS)** | 72 | 18 | -0.2 | -2.0 | 1.2 | 30 | 17 | -0.3 | -2.3 | 1.6 | 0.791 |
|  |  |  |  |  |  |  |  |  |  |  |  |
| **Between 6-10 months** |  |  |  |  |  |  |  |  |  |  |  |
| **Weight (SDS)** | 62 | 30 | 0.0 | -1.6 | 1.2 | 28 | 22 | 0.0 | -1.5 | 2.2 | 0.688 |
| **Length (SDS)** | 61 | 31 | 0.3 | -1.6 | 1.9 | 30 | 17 | 0.3 | -1.5 | 1.9 | 0.933 |
| **Head circumference (SDS)** | 62 | 30 | 0.2 | -1.4 | 1.5 | 28 | 22 | 0.0 | -1.7 | 2.3 | 0.777 |
| **BMI**^a^ **(SDS)** | 60 | 32 | -0.3 | -1.7 | 1.0 | 27 | 25 | -0.4 | -2.0 | 1.6 | 0.545 |
|  |  |  |  |  |  |  |  |  |  |  |  |
| **Between 11-15 months** |  |  |  |  |  |  |  |  |  |  |  |
| **Weight (SDS)** | 76 | 14 | 0.0 | -1.5 | 1.8 | 33 | 8 | 0.0 | -1.4 | 2.1 | 0.817 |
| **Length (SDS)** | 77 | 13 | 0.2 | -1.9 | 2.2 | 33 | 8 | 0.2 | -1.2 | 2.2 | 0.779 |
| **Head circumference (SDS)** | 77 | 13 | 0.0 | -1.5 | 1.6 | 32 | 11 | 0.0 | -1.7 | 1.6 | 0.782 |
| **BMI (SDS)** | 76 | 14 | 0.1 | -1.5 | 1.4 | 32 | 11 | -0.3 | -2.0 | 1.8 | 0.162 |
|  |  |  |  |  |  |  |  |  |  |  |  |
| **Between 16-20 months** |  |  |  |  |  |  |  |  |  |  |  |
| **Weight (SDS)** | 72 | 18 | 0.2 | -1.0 | 2.0 | 33 | 8 | 0.4 | -1.1 | 2.0 | 0.396 |
| **Length (SDS)** | 71 | 19 | 0.3 | -1.3 | 2.3 | 31 | 14 | 0.4 | -1.3 | 1.9 | 0.553 |
| **Head circumference (SDS)** | 70 | 20 | 0.2 | -1.7 | 1.2 | 31 | 14 | -0.1 | -1.7 | 1.7 | 0.524 |
| **BMI**^a^ **(SDS)** | 71 | 19 | 0.1 | -1.6 | 1.7 | 30 | 17 | 0.3 | -2.0 | 2.1 | 0.537 |
|  |  |  |  |  |  |  |  |  |  |  |  |
| **Between 21-25 months** |  |  |  |  |  |  |  |  |  |  |  |
| **Weight (SDS)** | 52 | 41 | 0.3 | -1.4 | 1.7 | 26 | 28 | 0.5 | -0.8 | 1.8 | 0.439 |
| **Length (SDS)** | 51 | 42 | 0.4 | -1.4 | 2.5 | 26 | 28 | 0.5 | -0.8 | 1.9 | 0.474 |
| **Head circumference (SDS)** | 50 | 43 | 0.0 | -1.5 | 1.5 | 23 | 36 | 0.2 | -1.5 | 1.4 | 0.560 |
| **BMI**^a^ **(SDS)** | 51 | 42 | 0.2 | -1.5 | 1.9 | 26 | 28 | 0.1 | -1.2 | 1.9 | 0.628 |
|  |  |  |  |  |  |  |  |  |  |  |  |
| **Weight gain (SDS)** |  |  |  |  |  |  |  |  |  |  |  |
| **Delta 0-2 months** | 77 | 13 | -0.2 | -1.3 | 1.2 | 35 | 3 | -0.1 | -1.4 | 1.7 | 0.596 |
| **Delta 2-12 months** | 69 | 22 | 0.2 | -0.9 | 1.6 | 32 | 11 | 0.0 | -1.0 | 1.7 | 0.252 |
| **Delta 12-24 months** | 50 | 43 | 0.2 | -0.6 | 1.0 | 25 | 31 | 0.3 | -0.2 | 0.9 | 0.399 |
|  |  |  |  |  |  |  |  |  |  |  |  |
| **Length gain (SDS)** |  |  |  |  |  |  |  |  |  |  |  |
| **Delta 0-2 months** | 78 | 11 | 0.9 | -0.6 | 2.3 | 35 | 3 | 1.0 | -0.7 | 2.0 | 0.418 |
| **Delta 2-12 months** | 71 | 19 | 0.1 | -1.2 | 1.5 | 33 | 8 | 0.2 | -1.7 | 2.0 | 0.615 |
| **Delta 12-24 months** | 50 | 43 | 0.0 | -0.7 | 1.4 | 25 | 31 | 0.1 | -1.1 | 1.4 | 0.732 |
|  |  |  |  |  |  |  |  |  |  |  |  |
| **Head circumference gain (SDS)** |  |  |  |  |  |  |  |  |  |  |  |
| **Delta 0-2 months** | 72 | 18 | 0.3 | -1.2 | 2.2 | 32 | 11 | 0.4 | -0.6 | 2.2 | 0.194 |
| **Delta 2-12 months** | 71 | 19 | -0.2 | -1.6 | 0.9 | 30 | 17 | -0.5 | -1.7 | 0.8 | 0.448 |
| **Delta 12-24 months** | 49 | 44 | 0.0 | -0.9 | 0.9 | 22 | 39 | 0.1 | -1.0 | 0.7 | 0.799 |
|  |  |  |  |  |  |  |  |  |  |  |  |
| **BMI**^a^ **gain (SDS)** |  |  |  |  |  |  |  |  |  |  |  |
| **Delta 0-2 months** | 77 | 13 | -0.5 | -1.9 | 1.2 | 34 | 6 | -0.7 | -2.4 | 1.9 | 0.725 |
| **Delta 2-12 months** | 69 | 22 | 0.3 | -1.1 | 1.9 | 31 | 14 | -0.2 | -1.3 | 1.5 | 0.059 |
| **Delta 12-24 months** | 49 | 44 | 0.5 | -1.1 | 1.4 | 24 | 33 | 0.7 | -0.4 | 1.2 | 0.240 |

NC-ICSI: Natural Cycle ICSI, c-ICSI: conventional ICSI, *p-values derived from Wilcoxon rank-sum (Mann–Whitney) tests, BMI: body mass index in (kg/m2), SDS: Standard deviation score, Kg: kilogram, cm: centimeter
